# Interannual variability of internal tides in the Andaman Sea: an effect of Indian Ocean Dipole

**DOI:** 10.1038/s41598-022-15301-8

**Published:** 2022-06-30

**Authors:** B. Yadidya, A. D. Rao

**Affiliations:** Centre for Atmospheric Sciences, Indian Institute of Technology Delhi, New Delhi, 110016 India

**Keywords:** Physical oceanography, Ocean sciences

## Abstract

A marginal sea in the north eastern Indian Ocean, the Andaman Sea, has been known for the presence of high-amplitude internal waves since the nineteenth century. In this study, we explored the interannual variations of the internal wave activity in this complex region. We found that the Dipole Mode Index, which represents the Indian Ocean Dipole (IOD), influences the circulation in the Andaman Sea, which in turn impacts its density stratification on interannual scales. Ocean Reanalysis System 5 data (1993–2018) is used to see an increasing trend in the sub-surface stratification, whereas it showed a decreasing trend in the near-surface waters. Numerical model simulations carried out from 2009 to 2018 have shown that the interannual variability in the generation of semidiurnal internal tides is governed by distinct parameters (tidal forcing and stratification) at different sites in different months. Enhanced upwelling (downwelling) is observed during positive (negative) IOD events. Sensitivity experiments conducted between extreme IOD events (2006 and 2016) revealed an increase in internal tide generation from positive IOD to negative IOD. Furthermore, a sharp decrease in local baroclinic dissipation is seen during negative IOD, increasing baroclinic flux into the Andaman Sea. An increase in the strength of positive IOD could lead to enhanced diapycnal mixing due to strong local dissipation, whereas an increase in the intensity of negative IOD could result in amplified propagation of internal waves.

## Introduction

Internal waves (IW) propagate along density gradients inside the ocean and are omnipresent^1–3^. Internal tides (IT), IW of tidal periodicity, are generated by the flow of barotropic tidal currents over topographic features such as continental slopes, seamounts, and submarine ridges^4–6^. IW play a prominent role in turbulence-driven diapycnal mixing^7,8^. The diapycnal nutrient fluxes that emerge from this upper ocean mixing are widely acknowledged as critical to stimulating phytoplankton growth and maintaining primary productivity^9–11^.

**Figure 1:**
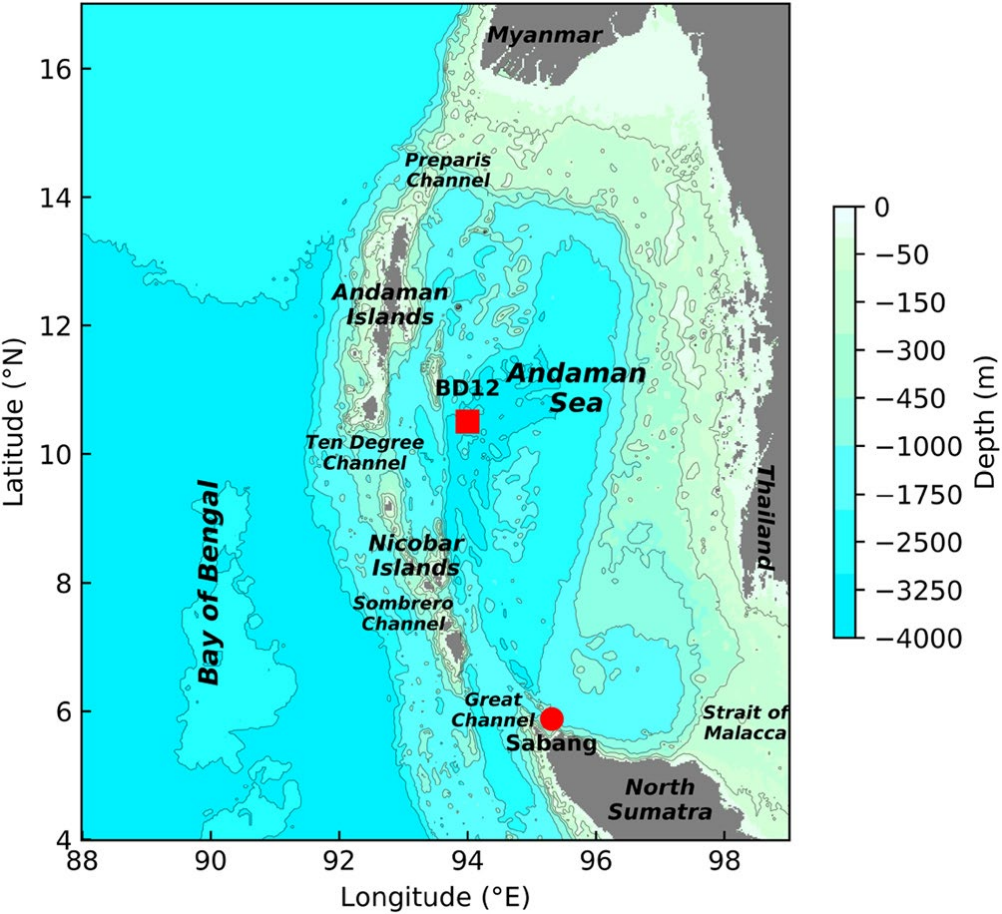
Model domain for the Andaman Sea along with the locations of BD12 and tide-gauge at Sabang. Bathymetry is derived from GEBCO. The relevant copyright information can be found at https://www.gebco.net/data_and_products/gridded_bathymetry_data/gebco_2019/grid_terms_of_use.html.

The Andaman Sea, a semi-enclosed marginal sea in the Indian Ocean, is known for its large amplitude IW. It is bounded by Malaysia and Thailand on the east, Sumatra Island on the south, and Myanmar on the north. On the west side, it is connected to the Bay of Bengal through Preparis Channel (PC), Ten Degree Channel (TDC), Sombrero Channel (SC), and Great Channel (GC) (Fig. 1). The bathymetry of the Andaman Sea varies drastically due to the volcanic origins of the Andaman and Nicobar Islands. It is quite distinct because of the multiple IW generation sites along the Andaman Nicobar Ridge, each with its own bathymetry and stratification^12^. IW had been seen long before the first scientific accounts of them were published as bands of choppy seas or ripplings stretching from horizon to horizon^13,14^. Later, remote sensing observations are used to identify and study the presence of IW^2,15–17^. Mohanty et al.^18^ quantified the IT energy budget using numerical modeling. However, the long-term variations in the IW activity and the cause for their variability is still unexplored territory.

Equatorial forcing primarily drives the mean coastal circulation in the Andaman Sea and around the Andaman and Nicobar islands^19^. The Indian Ocean Dipole (IOD) is the main parameter that drives the interannual variability in the equatorial Indian Ocean^20–22^. During positive IOD (pIOD), the warm sea surface temperature (SST) anomalies are seen in the western equatorial Indian Ocean, and cool SST anomalies are present in the east. In contrast, warm SST anomalies are observed in the eastern equatorial Indian Ocean during negative IOD (nIOD). A pIOD event typically begins to form in boreal summer by the generation of anomalous southeasterly winds and cooling off Sumatra-Java, and it peaks in boreal autumn (September, October, November). A local Bjerknes feedback^23^ occurs as the anomalous winds result in upwelling and lift the thermocline, which supports the initial cooling.

According to Baines^5^, the amount of energy converted from barotropic tide to IT is directly proportional to density stratification. Several experiments conducted using numerical model simulations also supported this theory and showed that increasing stratification led to an increase in IT generation by barotropic tide^23–27^. The phase speed and amplitude of IW are largely determined by density stratification^28^. Jithin et al.^29^ suggested that seasonal variability in stratification significantly altered the generation of IT in the Andaman Sea. Furthermore, Yadidya et al.^12^ found that the changes in energy conversion from barotropic tide to IT on the seasonal scale are mainly controlled by stratification. Therefore, we used density stratification as a proxy for IW activity in the Andaman Sea. The main goal of this paper is to identify the interannual variability of IW and the factors that control it in this region. Furthermore, to quantify the significance of stratification and tidal forcing for the observed variability in the generation of IT using model simulations. Finally, the difference in IT energy budget between pIOD and nIOD events is discussed.

## Data and methods

The Dipole Mode Index (DMI) with a rolling mean of 3 months is used to represent IOD and study its effects. It is an anomaly index of SST calculated from the difference in SST between the western (10° S-10° N, 50° E-70° E) and eastern (10° S-0° N, 90° E-110° E) regions of the tropical Indian Ocean. The index is based on the HadISST1.1 SST^30^ dataset analysis and retrieved from the NOAA/PSL website^31^. The anomaly is computed relative to a monthly climatological seasonal cycle based on the years 1981–2010. The model validation is carried out using density spectral estimate computed from *in-situ* temperature and salinity collected from BD12^32^.

The density stratification derived from Ocean Reanalysis System 5 (ORAS5)^33^ during 1993–2018 is analyzed to characterize the interannual variability of IW activity in the Andaman Sea (4–17° N, 92–99° E). Vertical temperature and salinity profiles from ORAS5 in each grid box are used to calculate buoyancy frequency (*N*). It is then reduced to two-dimensional monthly time series profiles by averaging the *N* values over the whole domain. The buoyancy frequency (density stratification) is defined as

1 $$N=\sqrt{\left(\frac{-g}{{\rho }_{0}}\right)\frac{\partial \rho }{\partial z}}$$

where $g$ is the gravitational acceleration, $\rho$ is the density calculated from ORAS5 temperature and salinity, ${\rho }_{0}$ is a reference ocean water density and $z$ is the depth.

The independent effect of temperature and salinity on density stratification is estimated by retaining one variable to its climatological mean and using the monthly time series of the other. The density stratification due to temperature changes (salinity changes), *N_temp* (*N_salt*), is obtained by using climatological monthly mean salinity (temperature) profiles and monthly temperature (salinity) profiles.

The ordinary least squares linear regression is used to test for significant trends of N with respect to time for seasonal time series. Furthermore, ordinary least squares are used to test for significant correlations between monthly N in the near-surface and sub-surface with the following time series: the DMI representing the IOD and mean sea surface height anomaly (SSHA) from ORAS5 describing the circulation in the Andaman Sea.

The linear baroclinic energy equation of IT, after neglecting the tendency and advection terms as a first-order approximation is

2 $$\langle Conv\rangle - \langle Di{v}_{bc}\rangle = \langle DI{S}_{bc}\rangle$$

where $Conv$ is the depth-integrated barotropic to baroclinic energy conversion, $Di{v}_{bc}$ is the depth-integrated baroclinic flux divergence, and $DI{S}_{bc}$ is the depth-integrated baroclinic tidal dissipation. The angle bracket denotes a 14-day average to nullify the influence of intratidal and neap-spring variability.

3 $$Conv=g{\int }_{-H}^{\eta }{\rho }^{^{\prime}}{w}_{bt}dz$$

4 $$DI{V}_{bc}={\nabla }_{h} \cdot \left[{\int }_{-H}^{\eta }{u}^{^{\prime}}{p}^{^{\prime}}dz \right]$$

where ${w}_{bt}$ is the barotropic vertical velocity, ${\uprho }^{^{\prime}}$ represents the density perturbation, ${u}^{^{\prime}}$ and ${p}^{^{\prime}}$ are the baroclinic components of tidal velocity and pressure perturbation, respectively. $H$ and $\upeta$ are the time-mean water depth and surface tidal elevation, respectively. The pressure perturbations are derived from the density anomalies based on Nash et al^34^

5 $${p}^{\mathrm{^{\prime}}}\left(z,t\right)=-\frac{1}{H}{\int }_{-H}^{\eta }{\int }_{z}^{\eta }g{\rho }^{\mathrm{^{\prime}}}\left(\widehat{z},t\right)d\widehat{z}dz+{\int }_{z}^{\eta }g{\rho }^{\mathrm{^{\prime}}}\left(\widehat{z},t\right) d\widehat{z}$$

The local dissipation efficiency^35,36^, $q$, is defined as

6 $${q}=\frac{{\int }_{{s}}{ds}\langle DI{S}_{bc}\rangle }{{\int }_{{s}}{ds}\langle Conv\rangle }$$

where ${\int }_{\mathrm{s}}ds$ represents the area-integration of respective variables.

### Model configuration

The three-dimensional Massachusetts Institute of Technology General Circulation Model^37^ (MITgcm) is used in this study. It is a hydrostatic/nonhydrostatic, z-coordinate finite-volume model which resolves the incompressible Navier–Stokes equations with Boussinesq approximation on an Arakawa-C grid. The model domain^38^ is bounded between 4° N–17° N and 88° E–99° E. The horizontal grid resolution in both zonal and meridional directions is 2.7 km. The model bathymetry is obtained from the General Bathymetric Chart of the Oceans^39^ (GEBCO) and is shown in Fig. 1. The vertical grid resolution is 5 m in the top 150 m and gradually decreases after that with a total of 48 levels. Along the bottom and lateral boundaries, no-slip and free-slip conditions are applied, respectively. The horizontal (vertical) eddy viscosity and diffusivity are parameterized with the help of the Smagorinsky formulation^40^ (K-profile parameterization scheme^41^). The bottom drag coefficient is assumed to be constant at 0.0025. A sponge layer of 0.25° thickness is imposed along each open boundary to minimize reflections and absorb waves that propagate out of the model domain^42^. As the semidiurnal frequency dominates the IW spectrum in this region^12^, the model is forced along all the open boundaries with the barotropic velocity of semidiurnal components (M_2_, S_2_) extracted from the TOPEX/Poseidon global tidal model (TPXO9.2)^43^. The temperature and salinity from ORAS5^33^ are used for model initialization.

## Results and discussion

### Interannual variability of density stratification

**Figure 2:**
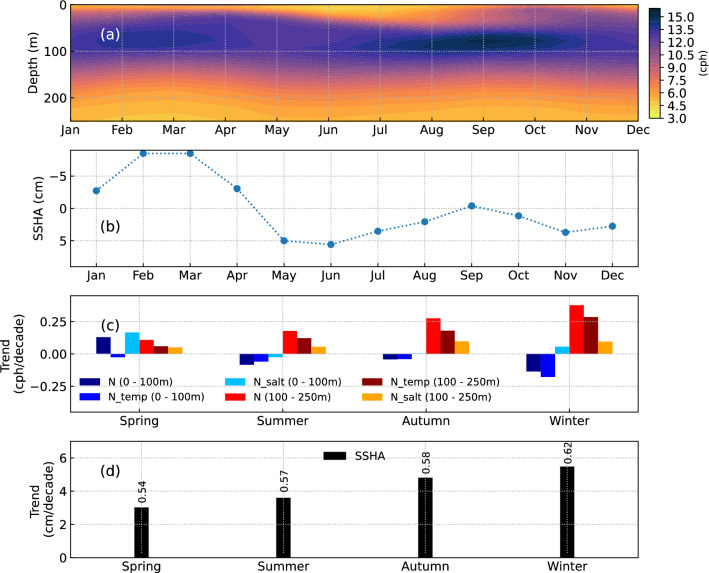
(**a**) Annual cycle of density stratification in the Andaman Sea. (**b**) Annual cycle of SSHA. (**c**) Linear trends of density stratification during different seasons. ‘N_temp’ and ‘N_salt’ represent the changes in stratification due to changes in temperature and salinity, respectively. (**d**) Linear trend of SSHA. The ‘R-value’ of linear trend is mentioned above the bars. Domain-averaged (4°–17°N, 92°–99°E) values derived from ORAS5 are considered in (**a**–**d**) to represent the Andaman Sea.

The annual cycle of the region averaged (4–17° N, 92–99° E) buoyancy frequency (Fig. 2a) displays a bimodal signal with two maxima in April and October. The near-surface (0 – 100 m) stratification is high during Spring and Autumn, whereas the sub-surface (100–250 m) stratification is maximum during Summer and Winter. The annual cycle of SSHA (Fig. 2b) shows the presence of upwelling waters from December to March and from June to September. On the contrary, downwelling is observed from March to June and September to December. A similar pattern is observed in the annual cycle of density stratification (Fig. 2a) and is probably the reason for its bimodal signal.

Seasonal stratification (Fig. 2c) in the sub-surface showed an increasing trend with the maximum increase during Winter. The near-surface stratification displayed a decreasing trend in all seasons except during Spring. Increasing stratification in the sub-surface arises predominantly from temperature changes, with salinity changes playing a secondary role. The temperature changes influence the decreasing trend in the near-surface except in Spring, where the increasing trend is due to salinity changes. SSHA (Fig. 2d) in the Andaman Sea showed an increasing trend of downwelling waters in all seasons. The increasing trend enhanced from Spring to Winter, which is similar to the trend observed in the sub-surface stratification. Therefore, we infer that the amplified downwelling is responsible for rising sub-surface stratification and declining near-surface stratification (except for Spring). The trend analysis describes the basic variability of circulation and stratification changes.

**Figure 3:**
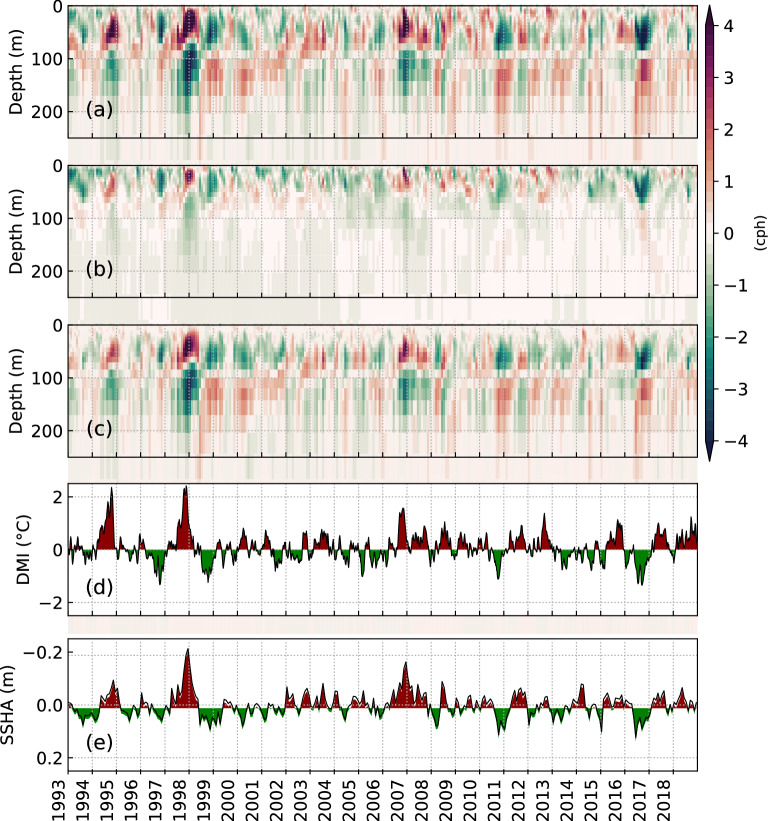
(**a**) Time-series of buoyancy frequency anomaly (after removing the annual cycle). (**b**) Time-series of buoyancy frequency anomaly due to salinity changes. (**c**) Time-series of buoyancy frequency anomaly due to temperature changes. (**d**) Time-series of DMI (3-month rolling mean) representing the IOD. (**e**) Time series of the detrended SSHA after subtracting the annual cycle. Domain-averaged (4°–17° N, 92°–99° E) values derived from ORAS5 are considered in (**a**–**c**) and (**e**) to represent the Andaman Sea.

The time-series of buoyancy frequency anomaly (Fig. 3a) after removing the annual cycle is compared with the following: time-series of buoyancy frequency anomaly only due to salinity changes (Fig. 3b) and only due to temperature changes (Fig. 3c); time-series of DMI (Fig. 3d), representing the IOD; and time-series of SSHA (Fig. 3e), representing the circulation in the Andaman Sea. The influence of salinity changes on the buoyancy frequency anomaly is generally seen in the upper 50 m (Fig. 3b). On the other hand, temperature changes were predominant below 40 m (Fig. 3c). A strong correlation is seen between positive (negative) anomalies of stratification in the near-surface (sub-surface) and pIOD (Fig. 3a,d). On the contrary, the negative (positive) anomalies of stratification in the near-surface (sub-surface) are observed during nIOD events. The correlation coefficient between DMI (Fig. 3d) and buoyancy frequency anomaly (Fig. 3a) in the near-surface (sub-surface) is 0.66 (− 0.60) with a delay of 2 months. The DMI (Fig. 3d) and SSHA (Fig. 3e) also displayed a strong correlation (− 0.62) with a time lag of 2 months. Strong upwelling is observed during pIOD events, and strong downwelling is observed during nIOD events. The correlation coefficient between SSHA and buoyancy frequency anomaly in the near-surface (sub-surface) is − 0.82 (0.89). Therefore, it is clear that the enhanced upwelling (downwelling) during pIOD (nIOD) leads to an increase in stratification in the near-surface (sub-surface) and vice versa.

The time series of buoyancy frequency anomaly and Niño 3.4 index, which represents El Niño–Southern Oscillation (ENSO), are compared in Supplementary Fig. S1 online. Earlier studies^44,45^ have suggested a relationship between SST in the Andaman Sea and ENSO. However, we found that the correlation coefficient between near-surface (sub-surface) stratification is 0.44 (− 0.51), which is relatively less than that of DMI. Therefore, the analysis in this study is constricted to the effect of IOD on IW. Nevertheless, ENSO could also be having a remote effect on the Andaman Sea stratification in conjunction with IOD. One such example is the pIOD event in 1997, along with El Niño resulted in strong buoyancy frequency anomalies (see Supplementary Fig. S1 online).

### Model simulations and validation

In the set of realistic simulations for April, 10 simulations are carried out from 2009 to 2018. The tidal forcing for the respective years is given from TPXO9.2, whereas the density stratification is derived from ORAS5 for the individual years and is inhomogeneous with respect to space. The model run starts on the 1st and continues until the 30th. The first two weeks of simulations are ignored as the model spin-up time to allow the IW generated in the Andaman Sea to propagate throughout the entire model domain and reach a steady-state near the farthest boundary^27,46,47^. The following two weeks (third and fourth week) are considered for analysis. The same procedure is repeated for October and December. These 3 months are chosen in this study because maximum depth-averaged stratification is seen during April and October, whereas the sub-surface stratification is relatively high in December.

**Figure 4:**
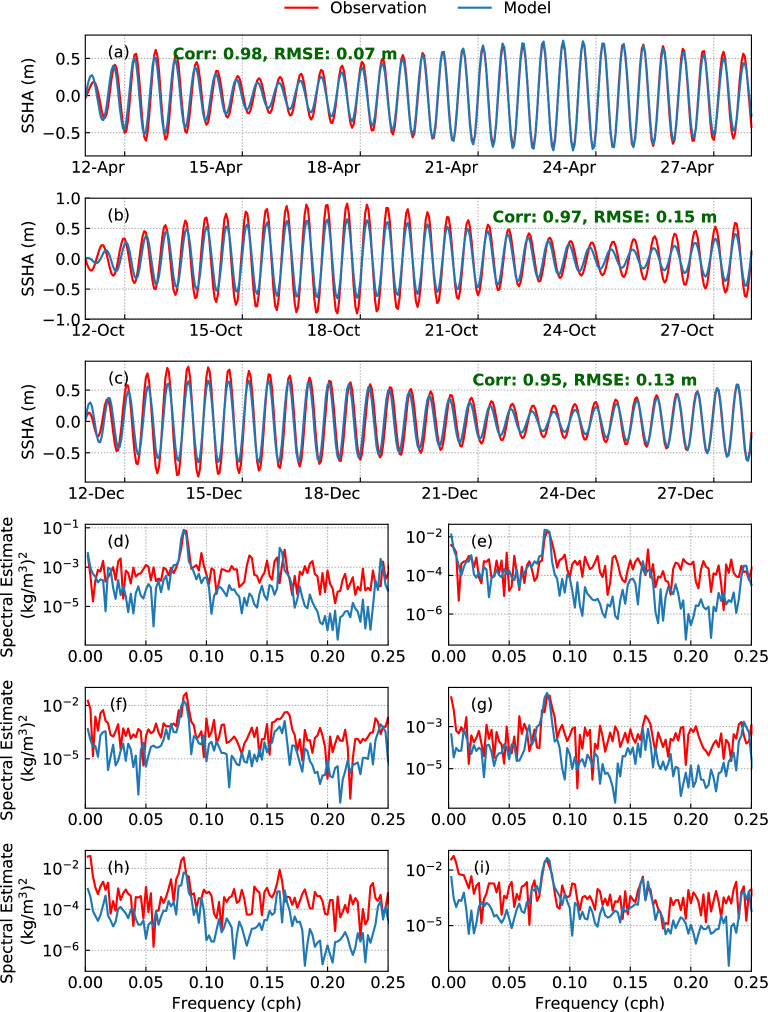
Comparison of model-simulated sea surface elevation anomaly with tide-gauge data at Sabang (5.88°N, 95.31°E) during (**a**) April 2016, (**b**) October 2016, and (**c**) December 2016. Comparison of the density spectral estimate between observations from BD12 and model simulations at (**d**,**f**,**h**) 75 m and (**e**,**g**,**i**) 100 m depth during (**d**,**e**) April 2016, (**f**,**g**) October 2016, and (**h**,**i**) December 2016.

The SSHA is compared between tide-gauge data at Sabang (5.88° N, 95.31° E) and model simulations during April (Fig. 4a), October (Fig. 4b), and December (Fig. 4c) of 2016. The correlation coefficient between the observations and model simulations is more than 0.95 in all 3 months. The root mean square error is 0.07 m, 0.15 m, and 0.13 m during April (Fig. 4a), October (Fig. 4b), and December (Fig. 4c), respectively. This validates the accuracy of the barotropic tidal signal in the model. Furthermore, the spectral estimate of density is compared between *in-situ* observations at BD12 and model simulations at 75 m (Fig. 4d,f,h) and 100 m (Fig. 4e,g,i). The model is able to capture the semidiurnal frequency M_2_ and its higher harmonics (M_4_, M_6_) reasonably well in all 3 months. The comparison of SSHA and spectral estimate of density are in good agreement for the remaining years whenever the observations are available but are not shown here.

**Figure 5:**
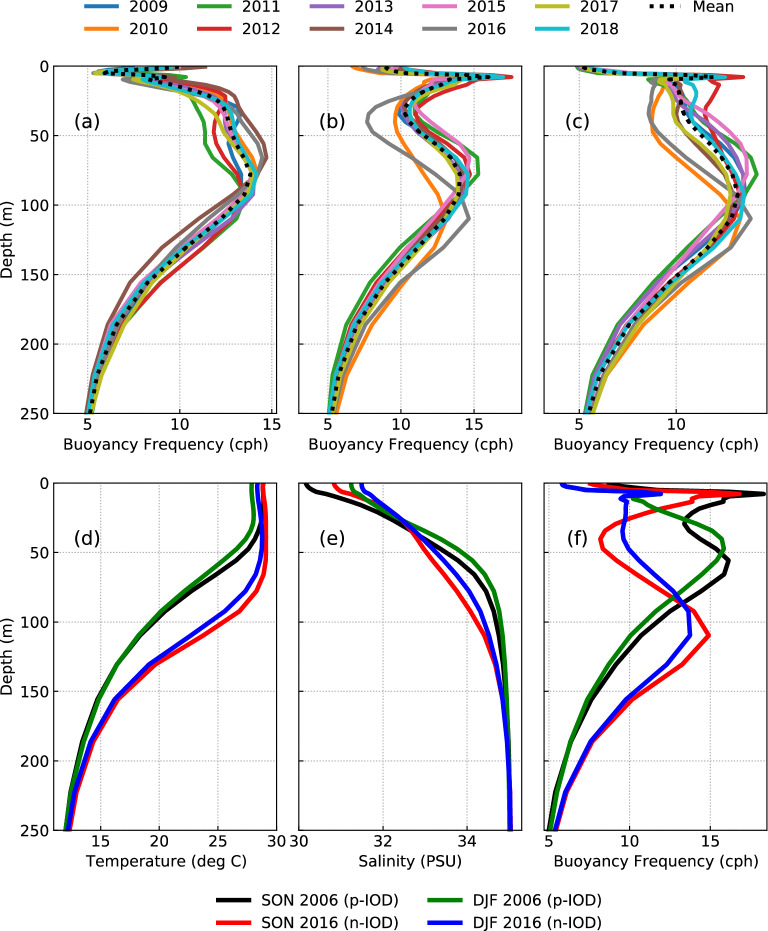
Mean buoyancy frequency profiles used in the set of experiments to quantify the effect of tidal forcing and stratification on the interannual variability of IT generation in (**a**) April, (**b**) October, and (**c**) December. Mean profiles of (**d**) temperature, (**e**) salinity, and (**f**) buoyancy frequency used in the set of experiments to study differences in IT generation, propagation, and dissipation between pIOD and nIOD. The legends for (**a**), (**b**), and (**c**) are shown on top, and the legends for (**d**), (**e**), and (**f**) are given on the bottom.

According to Song and Chen^48^, the variability of IT generation is caused by changes in astronomical tidal forcing and/or stratification. Therefore, to quantify the effect of tidal forcing on the interannual variability of IT generation, 10 simulations are performed for April by varying the tidal forcing and setting a constant homogeneous stratification. Time-averaged (ten-year mean of April) and domain averaged stratification profile from ORAS5 (Fig. 5a) is used.

Moreover, simulations are carried out by varying the stratification (Fig. 5a) and keeping a constant tidal forcing (April 2012) to quantify the effect of stratification on the interannual variability of IT generation. In this case, domain-averaged homogenous stratification for the respective years is used. Similar experiments are also carried out to understand the effect of tidal forcing and variable stratification for October (Fig. 5b) and December (Fig. 5c).

### Inter-annual variability in IT generation

**Figure 6:**
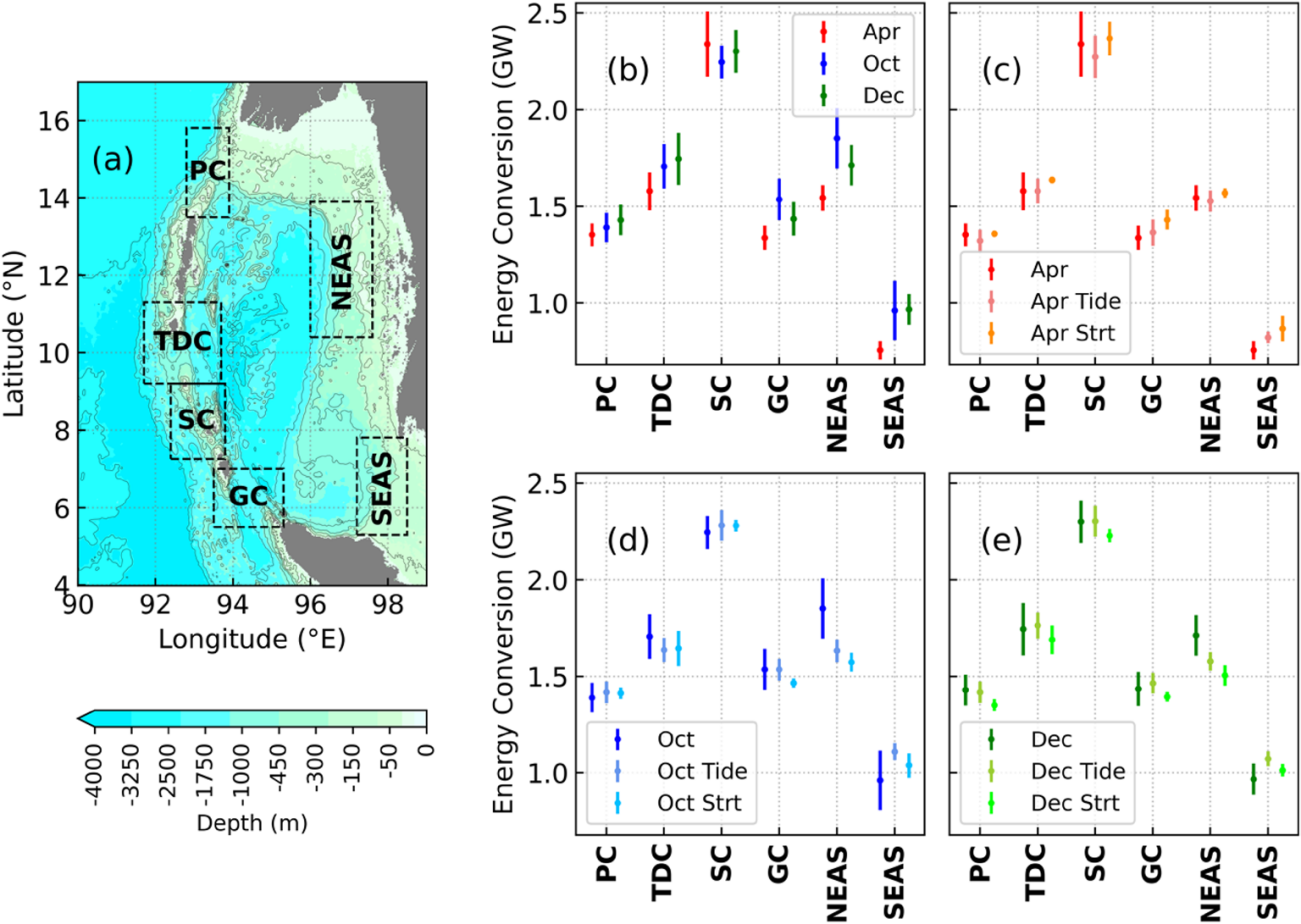
(**a**) Bathymetry of the Andaman Sea showing the sub-regions. (**b**–**e**) Standard deviation of semidiurnal IT energy conversion for different sets of experiments. ‘Apr’, ‘Oct’, and ‘Dec’ are set of realistic experiments. ‘Apr Tide’, ‘Oct Tide’, and ‘Dec Tide’ are set of experiments to quantify the effect of tidal forcing. ‘Apr Strt’, ‘Oct Strt’, and ‘Dec Strt’ are set of experiments to quantify the effect of stratification changes.

The sub-regions considered in the following analysis are shown in Fig. 6a. Figure 6b shows the mean and standard deviation of the energy conversion from the realistic set of simulations. In April, maximum IT generation (10 year mean of 2.34 GW) and high interannual variability (0.17 GW) are observed in SC. Minimum IT generation and the least interannual variance are noticed in the remaining regions. During October, maximum barotropic conversion and interannual variance are seen in GC (0.1 GW), North East Andaman Sea (NEAS) (0.15 GW), and South East Andaman Sea (SEAS) (0.15 GW). Whereas in December, the interannual variability is relatively high at PC (0.08 GW) and TDC (0.13 GW).

In Fig. 6c–e, the variance of the realistic set of simulations is compared with the set of simulations carried out to quantify the effect of tidal forcing and stratification in April, October, and December. Their mean density stratification profiles used to initialize the model simulations are shown in Fig. 5a–c for April, October, and December, respectively. The interannual stratification variability for the 10-years considered in this section is relatively higher during October and December than during April. In April, maximum variability is observed between 25 and 75 m. At these depths, the stratification is lower (higher) than usual in 2011 and 2012 (2014 and 2016), when the DMI is positive (negative). The maximum stratification is generally present near 75 m. Deepening (shoaling) of maximum stratification is seen in both October and December during nIOD (pIOD) events. However, the deepening is more profound than the shoaling. This could be due to the occurrence of relatively stronger nIOD events in the considered 10-year period. Maximum near-surface stratification is also seen at 10 m, more profoundly in October, due to a large influx of freshwater discharge into the Andaman Sea^49^. The maximum sub-surface stratification is somewhat deeper than April (80 m–95 m). However, the stratification between 25 and 50 m is relatively less in October and December than in April.

**Figure 7:**
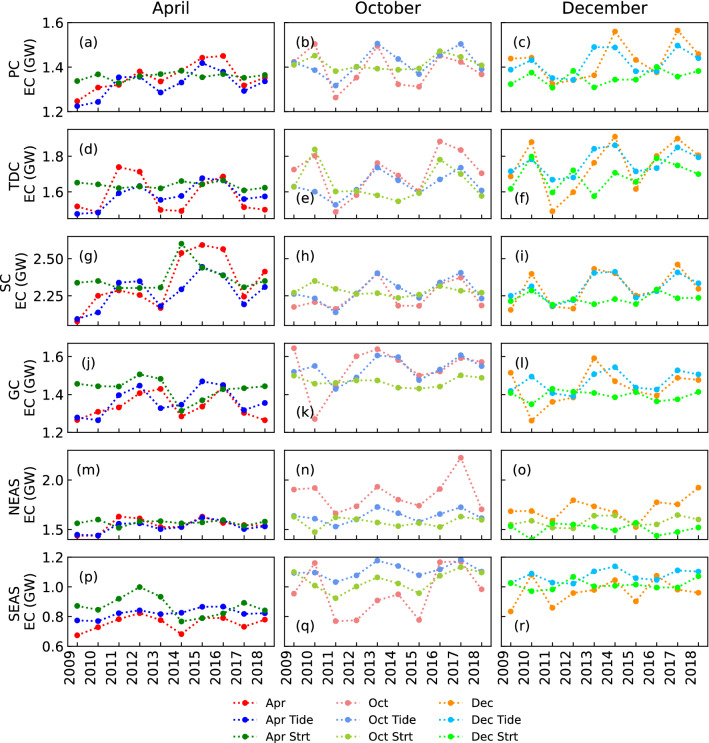
Interannual variability of semidiurnal IT energy conversion during April (**a**,**d**,**g**,**j**,**m**,**p**), October (**b**,**e**,**h**,**k**,**n**,**q**), and December (**c**,**f**,**i**,**l**,**o**,**r**) at PC (**a**–**c**), TDC (**d**–**f**), SC (**g**–**i**), GC (**j**–**l**), NEAS (**m**–**o**), and SEAS (**p**–**r**). ‘Apr’, ‘Oct’, and ‘Dec’ are set of realistic experiments. ‘Apr Tide’, ‘Oct Tide’, and ‘Dec Tide’ are set of experiments to quantify the effect of tidal forcing. ‘Apr Strt’, ‘Oct Strt’, and ‘Dec Strt’ are set of experiments to quantify the effect of stratification changes. EC on y-axis label means ‘energy conversion’.

In the PC, the changes in tidal forcing dominate the interannual variability in all the months (Fig. 7a–c), with the stratification variations playing a secondary role only in October (Fig. 7b) and December (Fig. 7c). Variability in the TDC is governed by stratification in October (Fig. 7e), and December (Fig. 7f) but is dominated by tidal forcing in April (Fig. 7d). For example, the high energy conversion in 2010 and 2016 during October (Fig. 7e) and December (Fig. 7f) is mainly due to high stratification. At SC and GC, stratification controls the interannual variability in IT generation in April (Fig. 7g,j), but tidal forcing plays a predominant role in October (Fig. 7h,k) and December (Fig. 7i,l). For example, the maximum (minimum) generation of IT in April 2014 (Fig. 7g,j) is due to an increase (decrease) in stratification in SC (GC).

The NEAS region is heavily influenced by freshwater flux, especially during Autumn^49^. Tidal forcing controls the variability in energy conversion in NEAS in April (Fig. 7m), with stratification playing a more dominant role in October (Fig. 7n) and December (Fig. 7o). In the SEAS region, changes in stratification are more critical in defining the amount of IT generated (Fig. 7p–r). It should also be noted that local stratification and sub-tidal currents^36,50–52^ could play a key role in both NEAS and SEAS. It could not be represented in the sensitivity experiments where homogeneous stratification is used. For example, the sudden increase in IT generation in October 2017 (Fig. 7n) at NEAS, which is not represented in both sets of sensitivity experiments, could be because of variations in local stratification^12^.

Stratification controls the interannual variability at SC, GC, and SEAS during April (Fig. 6c). Whereas in October (Fig. 6d) and December (Fig. 6e), stratification plays an important role at TDC, NEAS, and SEAS. Variations in astronomical tidal forcing control the interannual variability in the remaining locations. This clearly shows that the interannual variability in the amount of energy converted from barotropic tide to baroclinic tide is governed by different parameters at different locations in different months.

### Baroclinic tidal energy budget in contrasting pIOD and nIOD events

This section discusses the difference between pIOD and nIOD events in terms of IT generation, dissipation, and propagation in the Andaman Sea. The strongest pIOD (2006) and strongest nIOD (2016) in the last two decades are chosen for this study (Fig. 3d). The Autumn and Winter seasons of 2006 and 2016 are considered, and their region-averaged profiles of temperature, salinity, and density stratification derived from ORAS5 are shown in Fig. 5d–f, respectively. Four additional experiments are carried out with constant tidal forcing and varying the respective stratification for pIOD and nIOD in Autumn and Winter. The mixed layer depth during pIOD is as deep as 70 m, whereas during nIOD, it is seen only up to 35 m (Fig. 5d). This led to a steeper thermocline during nIOD below 80 m. During pIOD, a sharp halocline is present up to 75 m, whereas during nIOD, the halocline is deeper than 100 m (Fig. 5e). The density stratification (Fig. 5f) displays a bimodal structure in the vertical, with the first peak closer to the surface waters caused by the salinity gradient. The second peak, which is caused due to both temperature and salinity gradient, is observed at 55 m (45 m) in Autumn (Winter) during pIOD but dropped to 110 m (110 m) during nIOD. The density stratification during pIOD is very high in the near-surface, in this case up to 80 m, and decreases below that. During nIOD, the sub-surface stratification is very high, and the near-surface stratification is considerably less than that of pIOD. The main difference in stratification between Autumn and Winter is that the first peak due to the salinity gradient is strongest in Autumn. In contrast, the second peak is relatively stronger in Winter.

**Figure 8:**
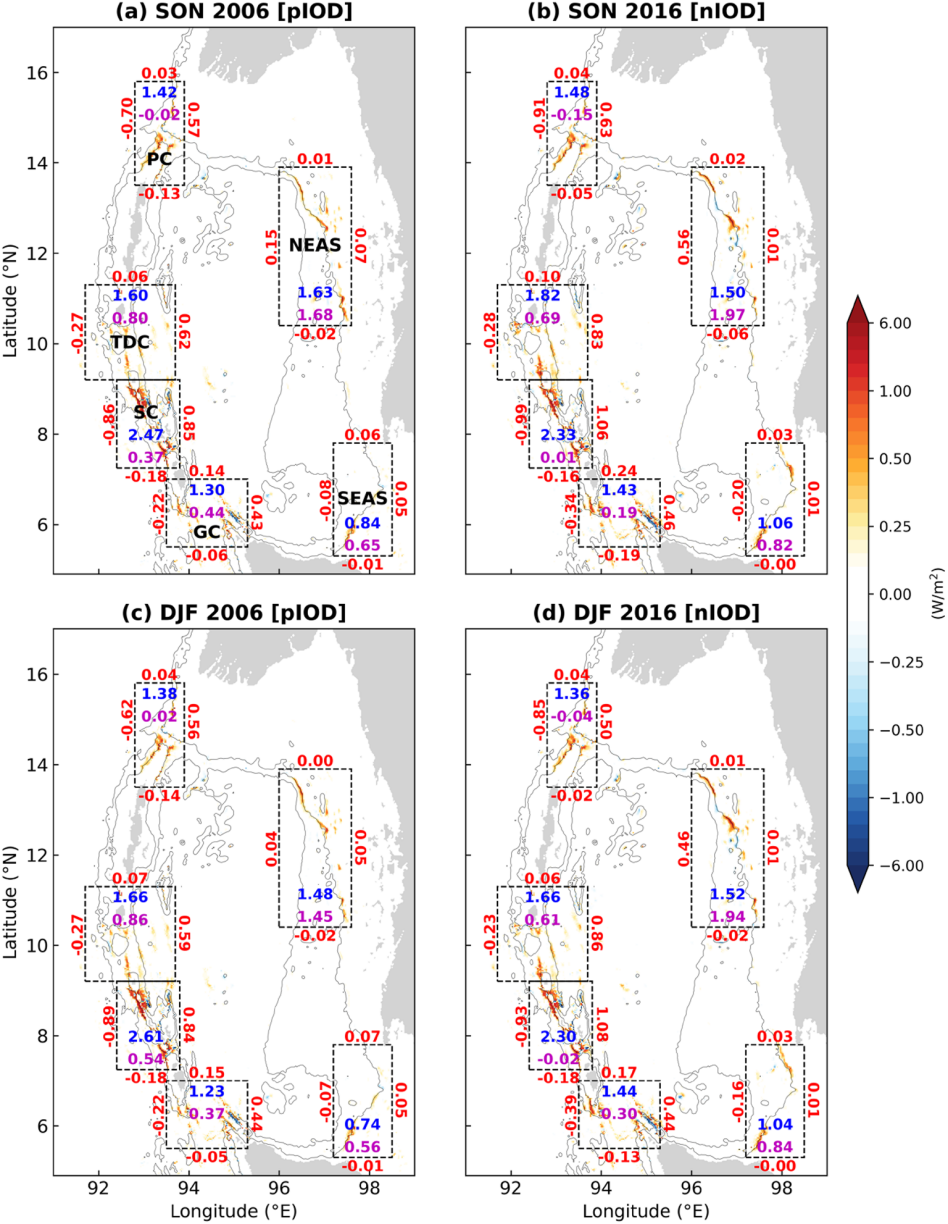
Comparison of baroclinic tidal energy budget between (**a**,**c**) pIOD and (**b**,**d**) nIOD during (**a**,**b**) Autumn and (**c**,**d**) Winter. The nonuniform color bar indicates the depth-integrated conversion rates from barotropic to baroclinic tide. The region-integrated and depth-integrated energy conversion (GW) and baroclinic dissipation (GW) are represented by blue and magenta numbers inside the box, respectively. Red numbers show the depth-integrated baroclinic energy fluxes (GW) along the boundaries.

During Autumn (Fig. 8a,b), at PC, the IT generation increased by 4.6% from pIOD to nIOD. The baroclinic flux increased by 30.6% and 11% in the west and east directions, respectively. In TDC, the baroclinic energy conversion increased by 14.2%, but the local baroclinic dissipation rate (*q*) decreased from 0.5 to 0.38. An increase in the IT propagation of 57% and 34% is noticed in the north and east directions, respectively. In the SC, which is the main generation site, the IT generation decreased by 5.5%. Baroclinic dissipation of 0.37 GW (local dissipation ratio of 0.15) is seen during pIOD, but during nIOD, almost all the generated IT propagated away from SC with very little baroclinic dissipation. A similar trend is observed in GC, where local dissipation efficiency decreased from 0.34 to 0.13 despite baroclinic conversion increasing by 10.5%. The decrease in baroclinic dissipation at TDC, SC, and GC resulted in more baroclinic flux entering the Andaman Sea and the Bay of Bengal, even more so into the former region. This can be seen along the western boundary of NEAS, where there is a sharp increase in the eastward propagating baroclinic flux from pIOD (0.15 GW) to nIOD (0.56 GW). Although there is a decrease in baroclinic conversion in NEAS, there is an increase in dissipation due to the dissipation of remotely generated IT. The maximum increase in the amount of IT generated (26.9%) and dissipated (27.1%) is seen in SEAS, where the westward propagation of baroclinic flux also increased from 0.08 GW to 0.2 GW.

The mean vertical profiles of temperature, salinity, and buoyancy frequency for multiple pIOD and nIOD events during Autumn are shown in Supplementary Fig. S2 online. The maximum sub-surface stratification during different pIOD (nIOD) is near 50 m (100 m). Therefore, the semidiurnal IT energetics could be similar to that of 2006 for pIOD events and 2016 for nIOD events, respectively, albeit with some minor differences in magnitude owing to the differences in the strength of IOD events.

During Winter (Fig. 8c,d), a similar trend of decreased local baroclinic dissipation at TDC, SC, and GC and increased propagation of IT into the Andaman Sea is observed from pIOD to nIOD even though its magnitude has differed. The local dissipation efficiency decreased from 0.52 to 0.37, 0.21 to 0, and 0.3 to 0.21, at TDC, SC, and GC, respectively. However, the IT generated at TDC remained constant during both the events, which was not the case during Autumn. The increase in IT generated in GC (16.9%) and SEAS (39.2%) is greater than it is in Autumn.

As reported by Kunze^53^, most of the dissipation near the IT generation sites occurs in the pycnocline. The differences in the local dissipation rates between pIOD and nIOD could result from differences in the main pycnocline depths. During pIOD, there could be more dissipation because of the shallow pycnocline interacting with more rough topography^54–58^ in the TDC, SC, and GC. It should be noted that the sub-tidal currents could significantly alter the propagation and dissipation of semidiurnal IT^36,50–52,59^. However, we are looking at the effect of IOD on IT in the absence of sub-tidal currents because uniform stratification is considered for model initialization.

## Conclusions

The annual cycle of stratification in the Andaman Sea showed a bimodal signal, with near-surface maxima during Spring and Autumn and sub-surface maxima during Summer and Winter. The circulation in the Andaman Sea (SSHA) is the primary reason for this phenomenon. The SSHA in all the seasons displayed an increasing trend in downwelling, which led to an increasing (decreasing) trend in the sub-surface (near-surface) stratification. Temperature changes dominated the increase in trend in subsurface stratification. Salinity changes played a dominant role only during Spring, when near-surface stratification showed an increasing trend (unlike other seasons). Since the equatorial forcing plays a major role in controlling the circulation in the Andaman Sea, we observed a good correlation between DMI and stratification. During nIOD (pIOD) events, a higher-than-normal downwelling (upwelling) led to an increase (decrease) in sub-surface stratification.

During April, the changes in tidal forcing play a primary role in controlling the interannual variability of IT energy conversion at PC, TDC, and NEAS, whereas the changes in stratification play a more critical role at SC, GC, and SEAS. During October and December, the interannual variability in IT generation is controlled by changes in tidal forcing in PC, SC, and GC. Stratification plays a predominant role at TDC, NEAS, and SEAS. The main difference in stratification from pIOD to nIOD is that the second peak dropped from 55 m (45 m) to 110 m (110 m) in Autumn (Winter). Local baroclinic dissipation decreased significantly, even though IT generation increased slightly from the pIOD to the nIOD event in both Autumn and Winter. This resulted in a sharp increase in the amount of IT propagating into the Andaman Sea.

In summary, if the current trend in increasing sub-surface stratification with increasing strength of nIOD events continues, it could increase the IT generation and propagation into the Andaman Sea. On the contrary, if the strength of pIOD events increases, it would result in more diapycnal mixing due to an increase in local baroclinic dissipation. The increase in the strength and frequency of IOD events can significantly impact the IW activity in the Andaman Sea.

## Supplementary Information

Supplementary Information.

## Data Availability

The reanalysis data from ORAS5 is downloaded from https://cds.climate.copernicus.eu/cdsapp#!/dataset/reanalysis-oras5. The data from BD12 is available upon request. The SSHA data at Sabang is downloaded from http://uhslc.soest.hawaii.edu/data/download/rq#uh123a.
