# Supplementary material for: Interannual variability of internal tides in the Andaman Sea: an effect of Indian Ocean Dipole
Source: Sci Rep. 2022 Jun 30;12:11104. doi: 10.1038/s41598-022-15301-8 (PMC9247166; doi:10.1038/s41598-022-15301-8)
Supplement: Supplementary file 1 — Supplementary Information. [file 41598_2022_15301_MOESM1_ESM.docx]

Interannual variability of internal tides in the Andaman Sea: an effect of Indian Ocean Dipole

**B. Yadidya^1*^, and A. D. Rao^1^**

^1^Centre for Atmospheric Sciences, Indian Institute of Technology Delhi, New Delhi-110016

*yadidyabadarvada@gmail.com

**Supplementary Information**

# Supplementary Data

The Niño 3.4 index with a rolling mean of three months represents ENSO. It is the area-averaged SST from 5° S - 5° N and 170° W - 120° W calculated from HadISST1.

Supplementary Figures

**Supplementary Figure S1.** (a) Time-series of buoyancy frequency anomaly (after removing the annual cycle). (b) Time-series of DMI (3-month rolling mean) representing the IOD. (c) Time series of Niño 3.4 index (3-month rolling mean) representing ENSO. Domain-averaged (4°-17° N, 92°-99° E) values derived from ORAS5 are considered to represent the Andaman Sea.

**Supplementary Figure S2.** Mean autumn profiles of (a) temperature, (b) salinity, and (c) buoyancy frequency during different pIOD and nIOD events derived from ORAS5 in the Andaman Sea (4°-17° N, 92°-99° E).
